# Models for temporal-spatial parameters in walking with cadence ratio as the independent variable

**DOI:** 10.1007/s11517-018-1919-8

**Published:** 2018-11-21

**Authors:** Juan Fang, Zaile Mu, Zhonghua Xu, Le Xie, Guo-Yuan Yang, Qiuju Zhang

**Affiliations:** 1 School of Mechanical Engineering, Jiangnan University, Wuxi City, 214122 Jiangsu Province China; 2 The Joint Lab of the Institute of Rehabilitation Engineering and Chejing Robotics Technology (Shanghai) Co., Ltd., Med-X Research Institute, Shanghai Jiao Tong University, Shanghai, 20030 China; 3 The Institute of Rehabilitation Engineering, School of Biomedical Engineering, Shanghai Jiao Tong University, Shanghai, 200030 China

**Keywords:** Temporal-spatial parameters, Stride length, Speed, Walk ratio, Cadence, Regression analysis

## Abstract

Accurate models that describe temporal-spatial parameters are desirable in gait estimation and rehabilitation. This study aimed to explore simple but relatively accurate models to describe stride length (SL), speed (SP) and walk ratio (WR) at various cadences. Twenty-four able-bodied participants (16 in a test group and 8 in a validation group) walked at seven cadence ratios (CRs). The individual and group mean SL, SP and WR were studied. Suitable temporal-spatial model structures were proposed and used to approximate the individual SL, SP and WR at various CRs. After the temporal-spatial model structures were found to be feasible, the general temporal-spatial models were analysed using the test group mean SL, SP and WR. Accuracy was assessed using the validation group mean values. Individual approximation accuracies showed that the proposed model structure deduced from the linear SL model was suitable for WR approximation. The linear, deduced quadratic and power functions approximated the individual SL, SP and WR, respectively, with high accuracy. Based on the test group mean SL, SP and WR, the general temporal-spatial models were obtained and produced comparable approximation accuracies in the validation group. The general temporal-spatial models predicted well the individual gait parameters with similar individual errors for both groups. These temporal-spatial models clearly describe SL, SP and especially WR at various cadences. They provide accurate reference data for gait estimation and have potential to guide speed modulation in robot-assisted gait rehabilitation.

**Electronic supplementary material:**

The online version of this article (10.1007/s11517-018-1919-8) contains supplementary material, which is available to authorized users.

**Graphical abstract:**
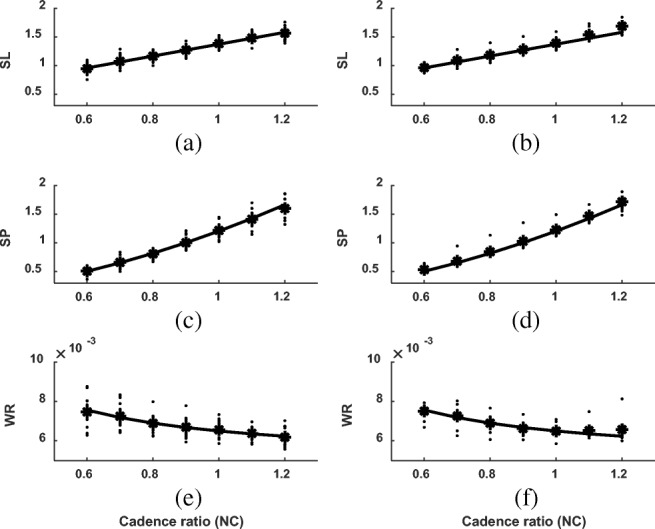
Twenty-four able-bodied participants (16 in test group and 8 in validation group) walked at seven cadence ratios (CRs), with the individual and group mean stride length (SL), speed (SP) and walk ratio (WR) studied. This work selected the cadence ratio as the independent variable and yielded general temporal-spatial models based on the test group data, which were a linear model for SL, a quadratic function for SP and a power function for WR. The general temporal-spatial model produced comparable approximation accuracies in the validation group. Clearly describing SL, SP and especially WR at various cadences, these temporal-spatial models provide accurate references for gait estimation and have the potential to guide speed modulation in robot-assisted gait rehabilitation. Approximation of the group mean temporal-spatial parameters at seven cadences. Solid lines in parts (a, b): the general linear SL model. Solid lines in (c, d): the general quadratic SP model. Solid lines in (e, f): the general WR model. Dots and stars in (a, c, e): the individual and group mean values for the test group. Dots and stars in (b, d, f): the individual and group mean values for the validation group.

## Introduction

In the field of gait estimation, it is important to understand the gait of able-bodied people while walking at a wide range of speeds. Human gait studies investigate temporal-spatial, kinematic and kinetic characteristics. Gait analysis provides important information for gait evaluation and guidance for gait rehabilitation programmes [1]. Temporal-spatial models describe the relationships between the biomechanical parameters of the lower extremities such as walking cadence, speed and stride length [2]. These gait parameters are often investigated at various speeds so as to provide reference data for analysis of pathological gait [3–7].

In addition to gait evaluation, temporal-spatial models are important for speed modulation in the gait robotics technology. These systems are required to assist walking at a wide range of speeds. Patients with gait impairments usually start to practise walking at a very low speed during the early post injury and/or operation phase [8]. Depending on the rate of recovery, endurance improvement, and individual training goals, the training speed gradually increases [9]. In order to physically challenge the patient and promote gait recovery, the training intensity increases even to a speed faster than the patient’s preferred gait velocity [10]. Within each training session, the gait robotic system is required to provide training at varying speeds. A slow walking speed is often used at the beginning and the walking speed then gradually increases to the target value [11]. In order to generate a natural gait pattern for smooth speed variation [12], the gait robotic system is required to modulate the cadence and stride length simultaneously [13–15]. Treadmill-based systems are further required to synchronise the movement of the gait robot and the treadmill surface [16–20]. Consequently, in the development and application of robotic-assisted gait rehabilitation, it is desirable to understand the gait patterns that able-bodied participants usually employ when walking at slow, normal and fast speeds.

Temporal-spatial patterns at different speeds have been extensively investigated [21–28], but as yet there is no general consensus. There has long been a strong desire to describe the gait parameters over a wide range of walking speeds. Since the 1950s, researchers have investigated the relationship between stride length, speed and cadence [21–23]. The empirical equations vary widely in form from constant [24], linear [25], quadratic [26], and hyperbolic [21] to more complex functions [22]. Furthermore, the parameters of these functions must vary from participant to participant to permit a satisfactory prediction of the individual temporal-spatial parameters. Different opinions also exist in regard to another gait parameter, walk ratio, which is the ratio of step length to cadence. Walk ratio was considered as a speed-independent index of walking patterns in some studies [24, 27], while it was reported to reduce with increasing speeds in other work [28, 29]. Furthermore, the mathematical description of WR has not yet been investigated. More research is required to clarify such research inconsistencies and limitations in the temporal-spatial relationship at various speeds.

Although walking speed significantly influences the gait pattern, it is not necessarily the direct factor that describes the temporal-spatial parameters. Speed is determined by stride length and cadence [26]. Different individuals control speed using different strategies including cadence adjustment only, stride length adjustment only or a combination of both [30, 31]. Such different strategies inevitably produce different joint kinematics even if people walk at the same speed. Therefore, some studies selected stride length [4] or cadence [32, 33] rather than walking speed as the independent variable to describe gait performance.

This study aimed to investigate simple but relatively accurate models to describe stride length, speed and especially walk ratio at various cadences. These functions can provide accurate reference data for gait estimation and have potential to guide speed modulation in robot-assisted gait rehabilitation.

## Methods

### Gait analysis experiment

As detailed in our previous study [33], a gait experiment was performed using a Vicon motion analysis system (Oxford Metrics Ltd., Oxford, UK) in Ruijin Hospital, Shanghai Institute of Orthopaedics and Traumatology, Shanghai, China. Ethical approval (Ref. No. 2013023) was obtained from the Ethics Committee at Med-X Research Institute, Shanghai Jiao Tong University, Shanghai, China. Twenty-four able-bodied participants (Table 1), with height ranging from 1.53 to 1.90 m, age from 18 to 63 years and body mass from 48 to 94 kg, were recruited to walk at seven different speeds. The speeds for each participant were controlled as follows: firstly, the participant chose his/her preferred normal walking speed. The cadence under these circumstances was recorded by a metronome as his/her individual normal cadence (NC). Then, the metronome beats were set to be 0.6, 0.7, 0.8, 0.9, 1.0, 1.1 and 1.2 times the individual NC. Finally, the participant was asked to walk barefoot along a 10-m walkway while following the predefined walking beats. The participants practised for 5 min to become familiar with the cadence control method before the data were recorded. The participants repeated each speed three times, giving a total of 504 trials. The actual cadence, strike length and speed were exported from the Vicon analysis software.

**Table 1:** Participant information. The lower limb length was the distance from the anterior superior iliac spine to the ipsilateral medial malleolus. *Px*, participant x; *NC*, normal cadence; *F*, female; *M*, male; *SD*, standard deviation; *NA*, not applicable. The participants marked with stars are from the validation group, while the rest are from the test group

Participant	Height (m)	Lower limb length (m)	Age (years)	Sex	Mass (kg)	NC (steps/min)	Stride length at NC
P1	1.53	0.83	60	F	58	100	1.29
P2	1.55	0.83	47	F	65	95	1.4
P3	1.57	0.83	54	F	62	110	1.52
P4	1.58	0.88	51	F	53	112	1.38
P5	1.61	0.85	51	F	65	105	1.29
P6	1.61	0.87	40	F	56	104	1.26
P7	1.64	0.84	32	M	84	110	1.52
P8	1.66	0.9	24	M	60	105	1.47
P9	1.66	0.9	50	F	63.4	110	1.34
P10	1.67	0.91	29	F	50	100	1.27
P11	1.72	0.93	22	M	72	105	1.49
P12	1.73	0.88	24	M	74	111	1.49
P13	1.74	0.93	63	M	65	110	1.29
P14	1.77	0.95	28	M	67	104	1.47
P15	1.81	1	31	M	77	111	1.36
P16	1.89	1.06	18	M	94	102	1.45
P17*	1.55	0.83	45	F	56	106	1.36
P18*	1.6	0.89	29	F	48	108	1.39
P19*	1.65	0.89	62	M	65	114	1.59
P20*	1.68	0.87	29	M	69	100	1.33
P21*	1.74	0.95	24	M	48	104	1.35
P22*	1.81	0.96	27	M	78	106	1.35
P23*	1.83	1	26	M	65	108	1.26
P24*	1.9	1.09	56	M	79	104	1.31
Mean ± SD	1.69 ± 0.11	0.91 ± 0.07	38.4 ± 14.5	NA	65.6 ± 11.5	106 ± 4.60	1.38 ± 0.09

This speed control method was examined for its test-retest reliability using an intraclass correlation coefficient (ICC). The actual NCs at three repetitions for all participants were analysed by calculating ICC and the 95% confidence interval (CI) for the ICC [34].

Stride length (SL), speed (SP) and cadence were directly obtained from the experimental data. Cadence ratio (CR) was recorded as the ratio of the actual cadence and the individual NC:

1 $$ \mathrm{CR}=\frac{\mathrm{cadence}}{\mathrm{NC}} $$

In this study, CR was set, as noted above, to 0.6, 0.7, 0.8, 0.9, 1.0, 1.1, and 1.2. It is known that temporal-spatial parameters SL, SP and cadence have an inherent relationship [1]:

2 $$ \mathrm{SP}=\frac{\mathrm{SL}\times \mathrm{CR}\times \mathrm{NC}}{120} $$

Furthermore, the temporal-spatial parameter walk ratio (WR) is defined as [24]

3 $$ \mathrm{WR}=\frac{\mathrm{SL}}{2\times \mathrm{CR}\times \mathrm{NC}} $$

Using the measured individual SL at various cadences, the experimental WR was calculated based on Eq. (3). The parameters SL, SP and WR hereafter refer to the values after normalisation by the individual lower limb length [35]. Using CR as the independent variable, the curves for the experimental SL, SP and WR were obtained for each participant.

### Analysis of suitable temporal-spatial model structures

In spite of intensive research on the parameter WR [24, 27–29, 36], a suitable approximation model for WR has never been investigated. In contrast, many models were proposed to describe SL and SP with respect to cadence, among which linear models were often adopted for SL [26, 28, 37, 38] and SP [1, 37]. Supposing NC is constant, and the actual cadence is normalised by NC (Eq. (1)), then the linear SL and SP models with respect to cadence can be described as

4 $$ \mathrm{SL}={a}_1\times \mathrm{CR}+{b}_1 $$

5 $$ \mathrm{SP}={a}_2\times \mathrm{CR}+{b}_2 $$

Using Eq. (2),

$$ {a}_1=0;{b}_2=0 $$

This shows that a linear model does not apply to both SL and SP simultaneously. Therefore, suitable model structures for SL, SP and WR approximation require to be determined.

Based on the often adopted SL and SP models, two assumptions can be made. If SL is approximated using a linear model (Eq. (4)), as proposed by studies [26, 28, 37, 38], then Eqs. (2)–(4) yield the models for SP and WR as

6 $$ \mathrm{SP}={a}_3\times {\mathrm{CR}}^2+{b}_3\times \mathrm{CR} $$

7 $$ \mathrm{WR}={a}_4+\frac{b_4}{\mathrm{CR}} $$

The other assumption is that SP is approximated using a linear model (Eq. (5)), as suggested by previous studies [1, 37]. From Eqs. (2), (3) and (5), SL and WR should take the following forms:

8 $$ \mathrm{SL}={a}_5+\frac{b_5}{\mathrm{CR}} $$

9 $$ \mathrm{WR}=\frac{a_6}{\mathrm{CR}}+\frac{b_6}{{\mathrm{CR}}^2} $$

Using the structures described in Eqs. (7) and (9), the experimental WRs at seven CRs from 24 participants were approximated. The fitness *R*^2^ (coefficient of determination) using both equations was calculated. During statistical analysis, unreasonable *R*^2^ values which were less than or equal to zero were not considered. Only those with 0 < *R*^2^ ≤ 1 from both equations were included to perform statistical analysis. A paired two-sided *t* test was used for hypothesis testing (normality of the data samples *R*^2^ was confirmed in both cases using the Kolmogorov-Smirnov test with Lilliefors correction), the null hypothesis being that no difference existed in the mean *R*^2^ from WR approximation using Eqs. (7) and (9). For the comparisons of means, mean difference (MD) and their 95% confidence interval (CI) were computed. The significance level for hypothesis testing was set to 5% (*α* = 0.05). Statistical analysis was carried out using the Matlab Statistics and Machine Learning Toolbox (The Mathworks Inc., USA).

### Feasibility analysis of the suitable temporal-spatial model structures

Using the model structures determined in Section 2.2 to approximate the temporal-spatial parameters, the individual accuracy *R*^2^ was analysed. If Eq. (7) was selected as the suitable structure for WR approximation, then linear regression analysis (Eq. (4)) was performed on the experimental SL from 24 participants. The deduced equations (Eqs. (6) and (7)) were respectively used to approximate SP and WR from 24 participants. Similarly, if Eq. (9) was determined as the suitable structure for WR, then linear regression analysis (Eq. (5)) was performed on the individual experimental SP, and the deduced equations (Eqs. (8) and (9)) were respectively used to approximate SL and WR from 24 participants. As no previous studies provided information on WR models, here, the acceptance criterion of the suitable temporal-spatial models was determined based on the literature [26], which was approximation of both SL and SP from 90% of participants with *R*^2^ > 0.90. If this criterion was not met, other models (e.g. second-order models) would be investigated.

### Analysis of general temporal-spatial models

In order to obtain and validate the general temporal-spatial models with CR, the participants were divided into two groups: a test group (16 participants) and a validation group (8 participants). The validation group was selected based on height: the participants whose heights were closest to 1.55, 1.6, 1.65, 1.7, 1.75, 1.8, 1.85 and 1.9 m were selected (marked with stars in Table 1). The test group means of SL, SP and WR at seven cadences were calculated. Using the model structures and approximation processes described in Section 2.3, the general approximation models for SL, SP and WR were obtained. The validation group means of SL, SP and WR at seven cadences were also calculated and were approximated using the corresponding models obtained from the test group.

The error in using the general temporal-spatial models to approximate individual experimental gait parameters was quantified using the root mean square error (RMSE):

10 $$ \mathrm{RMSE}(i)=\sqrt{\frac{1}{7}\sum \limits_{j=1}^7{\left({y}_{\mathrm{exp}}\left(i,j\right)-{y}_{\mathrm{approx}}\left(i,j\right)\right)}^2},i=\mathrm{1...24.} $$

where *i* represents the participant number, *j* represents the CR values on the range of 0.6–1.2, and *y*_exp_(*i*,*j*) represents the experimental SL, SP or WR from participant *i* walking at a cadence with CR index of *j*, while *y*_approx_(*i*,*j*) represents the corresponding approximated SL, SP or WR using the general temporal-spatial models. In order to allow inter-individual comparison, RMSE was normalised by the individual SL, SP and WR from walking at CR = 1.0.

## Results

### Accuracy of cadence control in gait analysis experiment

**Fig. 1:**
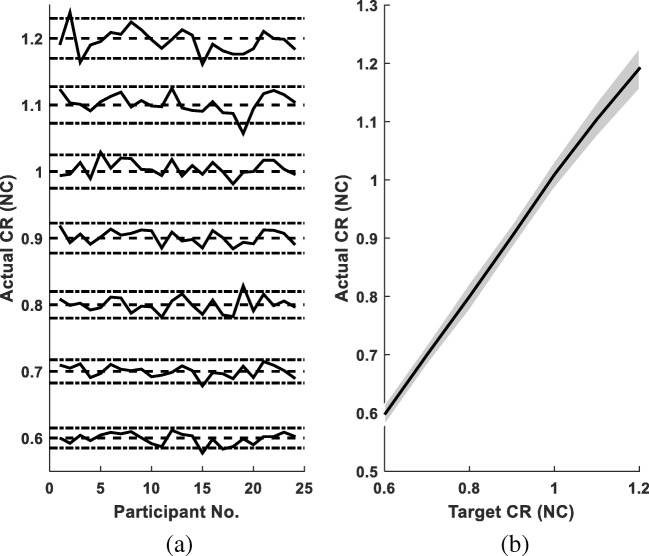
The actual CR. **a** The CRs (solid lines) of all 24 participants walking at seven cadences. A dashed line with two dash-dot lines in combination shows one target CR, and the area with an error less than 5%. **b** The mean CRs for all 24 participants. The solid line with shading represents experimental mean CR ± one SD

ICC for the three repetitions of NC for all participants was 0.89 with a 95% CI of (0.80, 0.95), which shows good reliability. The observed cadences of all participants were generally very close to the target cadences (Fig. 1). Exceptions were participant P15 walking at the targets of 0.6 NC, 0.7 NC and 1.2 NC; P19 at 0.8 NC and 1.1 NC; and P2 and P3 at 1.2 NC. Excluding these cases, all of the other walking sessions had cadences close to the target cadences with an error smaller than 5% (Fig. 1a). The mean cadences of all participants followed the target cadences closely with a standard deviation (SD) smaller than 0.035 NC (Fig. 1b). Such high reliability and such a small SD in the cadences from all participants show that the cadence control method via a metronome was feasible and reliable.

### Determination of suitable temporal-spatial model structures

**Fig. 2:**
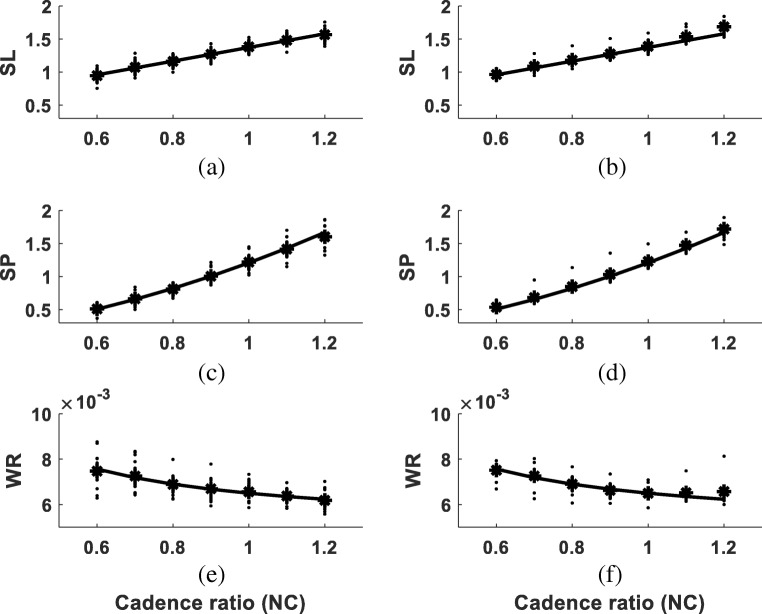
Approximation of the group mean temporal-spatial parameters at seven cadences. Solid lines in parts (a, b): the general linear SL model (Eq. (11)). Solid lines in (c, d): the general quadratic SP model (Eq. (12)). Solid lines in (e, f): the general WR model (Eq. (13)). Dots and stars in (a, c, e): the individual and group mean values for the test group. Dots and stars in (b, d, f): the individual and group mean values for the validation group

Individual SL and SP increased with cadence (dots in Fig. 2(a–d)). In contrast, WR slightly reduced with increasing CR (dots in Fig. 2(e–f)). Regression analysis showed that WR approximation using Eq. (9) produced nine unreasonable *R*^2^ values (*R*^2^ ≤ 0), while the nine corresponding *R*^2^ values from WR approximation using Eq. (7) were acceptable (mean ± SD 0.57 ± 0.25). Although statistical analysis of the remaining 15 *R*^2^ values failed to reject the null hypothesis of equal means in both cases (*p* = 0.13, Table 2), 10 out 15 *R*^2^ values from Eq. (7) were higher than Eq. (9). Therefore, Eq. (7) was determined as the suitable structure for WR approximation.

**Table 2:** *R*^2^ from WR approximation using Eqs. (7) and (9), and *p* value for comparison of means

Mean ± SD		MD (95% CI)	
Eq. (7)	Eq. (9)	Eqs. (7)–(9)	*p* value
0.85 ± 0.13	0.80 ± 0.16	0.049 (− 0.016, 0.11)	0.13

Notes: *n* = 15; Eq. (7) is WR model deduced from linear SL; Eq. (9) is WR model deduced from linear SP. *MD*, mean difference in *R*^2^ using Eqs. (7) and (9); *SD*, standard deviation; *CI*, confidence interval. 95% CI, 95% confidence interval for the mean difference; *p* value, paired two-sided *t* test

### Feasibility analysis of the suitable temporal-spatial model structures

Linear regression analysis of the individual experimental SL curves showed that 22 out of 24 participants had their SL well approximated with *R*^2^ > 0.9. *R*^2^ overall was 0.97 ± 0.034 (mean ± SD). Approximation of the individual SP curves using the deduced Eq. (6) showed that all participants had their SP well approximated with *R*^2^ > 0.95. *R*^2^ overall was 0.98 ± 0.012. Such high accuracies of SL and SP approximation met the feasibility criterion, which proved that it was feasible to use a linear model and a deduced quadratic function, respectively, to approximate SL and SP. Regression analysis of the individual WR curves using the deduced Eq. (7) showed that seven participants had their WR well approximated with *R*^2^ > 0.90. *R*^2^ overall was 0.72 ± 0.26.

### Determination of general temporal-spatial models

The test group means SL, SP and WR while walking at seven cadences were calculated (stars in Fig. 2(a, c and e)). Linear regression analysis yielded the general best-fit model for SL as

11 $$ \mathrm{SL}=1.0353\times \mathrm{CR}+0.3368,{R}^2=0.9980. $$

The linear model (solid line in Fig. 2(a)) fit the experimental means SL of the test group (stars in Fig. 2(a)) very well. Using Eqs. (2), (3) and (11) yielded the approximation functions as well as the approximation accuracies *R*^2^ for SP and WR:

12 $$ \mathrm{SP}=0.9107\times {\mathrm{CR}}^2+0.2963\mathrm{CR},{R}^2=\mathrm{0.9951.} $$

13 $$ \mathrm{WR}=0.0049+\frac{0.0016}{\mathrm{CR}},{R}^2=0.9841. $$

Equations (12) (solid line in Fig. 2(c)) and Eq. (13) (solid line in Fig. 2(e)) fit the experimental mean SP (stars in Fig. 2(c)) and WR (stars in Fig. 2(e)) from the test group very well.

Using Eqs. (11)–(13) to respectively approximate the validation group mean SL (stars in Fig. 2(b)), SP (stars in Fig. 2(d)) and WR (stars in Fig. 2(f)) yielded *R*^2^ as 0.96, 0.99 and 0.84.

**Fig. 3:**
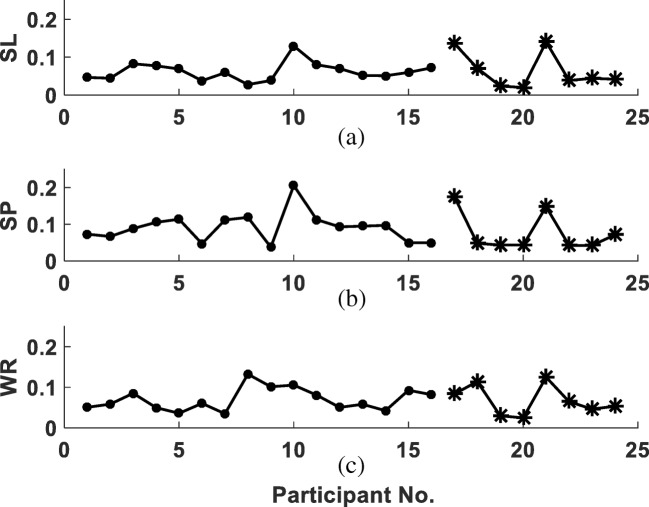
RMSE between the general temporal-spatial models and the individual experimental values. Dots: the test group. Stars: the validation group

The general temporal-spatial models predicted well the individual experimental SL, SP and WR (Fig. 3). The individual errors using the general model were comparable for both groups: the mean RMSE for SL, SP and WR is respectively 6.2%, 9.2% and 7.0% for the test group, and 6.5%, 7.7% and 6.8% for the validation group. The total mean temporal-spatial RMSE for all participants was 7.3%.

## Discussion

Using experimental gait data from 24 participants walking at seven cadences, this study explored simple but relatively accurate models to describe stride length, speed and walk ratio at various cadences. These functions can provide accurate reference data for gait estimation and have potential to guide speed modulation in robot-assisted gait rehabilitation.

One innovation of this study was to use CR as the independent variable to analyse the temporal-spatial parameters at various speeds, which reduced inter-individual variability. Most gait parameters are correlated to age, height and body mass [39], but NC is a fairly constant parameter [2, 40]. Therefore, this study designed the gait experiment and analysed the gait parameters using CR as the independent variable. After NC was determined for each participant, he/she was instructed to control walking speed by the metronome beats which were adjusted by the experimenter. Therefore the participant achieved walking at different CRs, i.e. the cadences normalised by their individual NC. This speed control method was adopted for three reasons: (i) there were many previous studies [28, 30, 41] which used metronomes to regulate the speed well; (ii) cadence control provided the possibility of comparing gait patterns at various speeds among groups with different anthropometric characteristics; and (iii) metronome guidance helped the participants to adopt similar strategies to change speed, i.e. cadence modulation [31]. In this study, the metronome control method showed good reliability (ICC = 0.89). Furthermore, all participants walked at the target cadences with a small SD of 0.035 NC. Therefore, the cadence control method via a metronome in this study was deemed feasible for speed control.

This work obtained simple and feasible temporal-spatial model structures. Accurate temporal-spatial models have always been desirable in the biomechanical field [21–28]. However, it can be difficult to obtain correct mathematical models because the temporal-spatial parameters are easily influenced by factors such as age [42], floor conditions [43, 44], and subjective attention [45]. Therefore, simple empirical models are often used instead [1, 26, 28, 37, 38]; these describe the gait parameters within a certain speed range with a certain accuracy. In order to obtain empirical models based on experimental data, it is vital to choose appropriate model structures. Linear models are often adopted, as seen in the description of SL [26, 28, 37, 38] and SP [1, 37]. Those linear approximations were considered acceptable for their purpose. However, this study demonstrated that a linear model was more suitable for SL (Eq. (4)) than for SP (Eq. (5)), after taking WR approximation into consideration. In WR approximation, Eq. (9) (deduced from Eq. (5)) produced 9 unreasonable *R*^2^ values. In the remaining 15 paired samples, 10 obtained lower *R*^2^ using the model Eq. (9) than Eq. (7) (deduced from Eq. (4)). Therefore, Eq. (5) was rejected in favour of Eq. (4). Furthermore, the individual SL curves were well approximated by the linear model Eq. (4) (*R*^2^ > 0.9 in 22 out of 24 participants). The deduced quadratic model Eq. (6) also well approximated the individual SP curves (*R*^2^ > 0.95 in all participants). WR, which is the ratio of step length to cadence, is expected to have higher variability than stride length. Nevertheless, WR approximation using Eq. (7) showed a mean accuracy of *R*^2^ = 0.72. It is possible that other temporal-spatial models exist with higher accuracies if more complicated structures are used. However, the model structures used in this study were considered as satisfactory empirical models, because (i) they were developed based on the simple but often adopted linear SL model and (ii) they produced accuracies high enough to meet our acceptance criterion. Therefore, the acceptable model structures for SL, SP and WR were finally determined as the structures described in Eqs. (4), (6) and (7), respectively.

Determination of model structures provided the basis for estimation of the general temporal-spatial models, but application of these general models requires data preprocessing techniques. Using the determined model structures, the test group mean values yielded general models for SL (Eq. (11)), SP (Eq. (12)) and WR (Eq. (13)) with high accuracies of approximation of *R*^2^ > 0.98. These models also produced comparable accuracies in the approximation of the validation group. Furthermore, these models predicted well the individual gait parameters, with a total mean temporal-spatial RMSE of 7.3%. It should be noted that these models only hold true when the following three conditions are met: (i) CR is used as the independent variable, which is the cadence normalised by NC; (ii) NC is constant; and (iii) the parameters SL, SP and WR are normalised by lower leg length. A comfortable walking pace NC is subject to an individual’s interpretation [2]. It varies slightly when the participant adopts different walking modes [28] or is in different concentration states [45]. Special attention should be paid to NC when it is measured before using the general temporal-spatial models.

Although the literature supports our approach of using a linear model to describe SL at various cadences, the applicable cadence range requires further investigation. The linear SL model in the current study was consistent with many previous studies [37, 38, 46]. However, there are studies [26, 28] which failed to obtain a satisfactory linear model for SL in cadence-controlled walking. In fact, study [28] showed a positive linear relationship between SL and cadence in the cadence mode, although the slope was small and difficult to observe. Egerton et al. reported that only 11% of participants showed a linear relationship between SL and cadence [26]. It was proposed that a linear model could only be used to approximate SL within a certain range of walking cadences [46]. If the cadence was higher than the maximal value, which is called the break point, SL would remain the same or decrease. Morris et al. set the break point as 129 steps/min and obtained a well-fit linear model for SL when cadence was lower than the break point [46]. Similarly, in the current study, the participants walked at 57–137 steps/min, and the cadence from 98% of the walking sessions was lower than 129 steps/min. A linear model for SL was obtained with a high accuracy (mean *R*^2^ = 0.97). In contrast, study [26] analysed walking sessions with cadence up to 150 steps/min, therefore; it was not surprising that a linear model failed to give a satisfactory accuracy for SL in that study. In summary, the linear SL model is generally well supported, although the cadence range for its application requires further research.

The WR model increases our knowledge of normal gait patterns and can improve the usage of WR in pathological gait estimation. It is believed that this was the first investigation regarding the mathematical WR model. WR was previously considered invariant over a wide range of speeds [24, 27]. The preferential WR was thought to be close to the value that minimised the energy cost for normal gait [47]. A decrease in WR was thought to be related to a reduced step length and/or increased cadence, thus WR was used as a detection index of pathological gait [36]. In contrast, some studies [28, 29] obtained different results for WR. Zijlstra et al. observed a changeable WR when the walking conditions were different [28]. The gait data in the current work agree with study [28] that WR reduces with increasing cadence. It is proposed here that the inconsistency between the current study and [24] came from the fact that the lowest cadence the participants in [24] walked at (76.6 steps/min) was not as low as here (57 steps/min). Therefore, the reducing trend of WR in study [24] was not easily observed. In fact, Sekiya et al. observed that WR was variant “at lower speeds” and also reported a reduction, albeit slight, in WR with increasing speeds in the female group [24]. Since WR was not as changeable as speed, study [24] concluded that WR was “constant at moderate speed”. WR variability at low speeds was further explored in a recent study [29] which observed that when the walking speed decreased, WR and its variance began to increase abruptly. WR model Eq. (13) accurately explained the observation that WR reduced with increasing cadence and reduced more rapidly at lower cadences. Such WR variation with speed should be considered when using WR to evaluate gait performance, judging by the fact that most pathological gaits occur at low speed.

Apart from gait estimation, these simple and relatively accurate general temporal-spatial models can be used to guide speed modulation in robot-assisted gait rehabilitation. During gait training, therapists and/or patients usually define the speeds to be employed. However, regarding the gait robotic system, how fast it moves is mainly defined by the cadence and SL produced by the gait orthosis. Using the obtained SP model, the target cadence for the prescribed speed can be calculated. Then using the SL model, the corresponding stride length can be obtained to achieve the specific training speed. Such SL/SP models can be used to determine the target cadences and stride lengths for various speeds prescribed by the users of gait robotics.

Future work will focus on further investigation of the temporal-spatial models regarding the applicable cadence range and re-evaluation of the model parameters using more gait data. A limitation of this study was that the temporal-spatial models were deduced based on data walking at cadences between 0.6 and 1.2 NC, which was 57–137 steps/min. The cadence range within which these temporal-spatial functions are valid needs further investigation. Another limitation was that the general temporal-spatial models were obtained based on data from participants with large age and mass ranges. This study specifically recruited participants with widely varying anthropometric characteristics, so that the determined model structures are generally applicable. To eliminate the influence of height, the models in the current study used gait parameters that were normalised by lower limb length. However, age and body mass inevitably influenced the actual model parameters. Further studies using more participants from a smaller age/mass range are planned to further re-evaluate the parameters for the general temporal-spatial models.

## Conclusions

Using CR as the independent variable, this study investigated simple temporal-spatial models structures, which was proven feasible using gait data from 24 able-bodied participants. Instead of being a speed-independent constant parameter, WR reduced slightly with increasing cadence and reduced faster at lower cadence. Using group mean data, this study obtained and validated a robust linear model for SL, a quadratic function for SP, and a power function for WR for walking at 0.6–1.2 NC. Although the exact parameters of the general temporal-spatial models require further re-evaluation using data from more participants walking at a wider speed range, these models increase knowledge of normal gait patterns and can be applied in gait estimation and robot-assisted gait rehabilitation.

## Electronic supplementary material

ESM 1 (MAT 6 kb)

ESM 2 (M 964 bytes)

## Abbreviations

CI: Confidence interval
CR: Cadence ratio
ICC: Intraclass correlation coefficient
MD: Mean difference
NA: Not applicable
NC: Normal cadence
RMSE: Root mean square error
SD: Standard deviation
SL: Stride length
SP: Speed
WR: Walk ratio
